# A multi-method feasibility trial of a multi-component behaviour change intervention to reduce sedentary behaviour and increase physical activity among ethnically diverse older adults

**DOI:** 10.1136/bmjopen-2024-084645

**Published:** 2024-11-07

**Authors:** Naureen Akber Ali Meghani, Joanne Hudson, Gareth Stratton, Jane Mullins, Deepak Sahoo

**Affiliations:** 1 Swansea University College of Engineering - Bay Campus, Swansea, UK; 2 Swansea University - Singleton Park Campus, Swansea, UK; 3 Swansea University School of Mathematics and Computer Science- Bay Campus, Swansea, UK

**Keywords:** Aged, Health Education, Primary Prevention, Aged, 80 and over

## Abstract

**ABSTRACT:**

**Introduction:**

Evidence suggests that sedentary behaviour (SB) and physical activity (PA) are important indicators of well-being and quality of life in older adults (OAs). However, OAs are the least active and highly sedentary of all the age groups. The present study intends to examine the feasibility of a wearable gadget to remind users to break sitting time (by standing up and moving more), coupled with a brief health coaching session, pamphlet and reminder messages to decrease SB and improve PA.

**Methods and analysis:**

This study will employ a multi-methods approach that generates quantitative data from questionnaires and qualitative data from semi-structured interviews following OAs’ involvement in the study. This intervention will be informed by the socio-ecological model (SEM) and the habit formation model. The quantitative and qualitative data will be analysed separately and then integrated for interpretation and reporting, which will assist our knowledge of the feasibility of the programme.

**Ethics and dissemination:**

Ethical approval for this study has been obtained from Swansea University (NM_ 2023 6667 6123). Informed consent will be obtained from participants. The findings of the study will be disseminated to the scientific community through conference presentations and scientific publications. The findings of the current study will determine the suitability of a future effectiveness trial.

**Trial registration number:**

NCT06407557.

**STRENGTHS AND LIMITATIONS OF THIS STUDY**

- The current research will include multi-ethnic OAs (≥65 years) who are under-represented in research, allowing the findings to be generalised beyond the non-Black, Asian and Minority Ethnic OA population.
- Sedentary behaviour will be assessed via a self-reported (Measure of Older Adults’ Sedentary Time) tool rather than an objective measure, which limits the accurate assessment of sedentary behaviour.
- The design does not include a control group, thus not allowing outcome comparisons between groups of participants who have and have not received the intervention.
- It is difficult to draw conclusions on the long-term impacts of the intervention due to the lack of follow-up assessments incorporated to measure adherence to the intervention.

## Introduction

 The world’s population is rapidly ageing as life expectancy increases.^1 2^ The promotion of healthy ageing, that is, the process of developing and sustaining functional capacity that supports well-being in older age,^3^ means that older adults (OAs) are more likely to engage actively and contribute to cultural, social, economic and other important life domains.^4^ It is estimated that healthy ageing can lower the prevalence of non-communicable diseases^5^ and extend life expectancy by 0.68 years.^6^ Studies have shown that for healthy ageing, it is essential to complete an adequate level of physical activity (PA).^6 7^ PA has been defined as any skeletal muscle-driven movement of the body that involves energy expenditure.^8^ There is significant and consistent evidence that regular engagement in PA is linked with physical and mental health benefits.^9^ According to recommendations from the WHO, OAs (aged ≥60 years) should take part in 150–300 min per week of moderate-intensity aerobic activity or 75–150 min per week of vigorous-intensity aerobic activity, or an equivalent combination of moderate-intensity and vigorous-intensity aerobic activity. In addition, on 2 or more days per week, they should perform muscle-strengthening and balance activities (such as strength and resistance training and yoga) to maintain health and well-being.^8^ However, many OAs do not participate in PA at the intensity, duration or frequency needed to improve their physical and mental health.^10 11^

Evidence suggests that in addition to PA, sedentary behaviour (SB) is an essential indicator of well-being and quality of life in OAs.^12^ SB refers to any waking activity characterised by an energy expenditure of ≤1.5 metabolic equivalents, while in a sitting, reclining or lying position.^13^ OAs are identified as one of the most sedentary groups in the population, who spend 70.1% of their time being sedentary at home,^14^ and 67% of OAs spend more than 8.5 hours per day being sedentary.^15 16^ There is increasing evidence to suggest that SB is detrimental to physical health^17^ and is correlated with a higher risk of all-cause and cardiovascular disease mortality, type 2 diabetes^18^ and lower physical and functional quality of life.^19^ In addition, a high level of SB is negatively associated with cognitive function, functional status, depression and disability in OAs,^20^,^22^ impacting healthcare costs.^23 24^ Moreover, SB is related to a lower likelihood of successful ageing.^25^ Currently, there are no recommended guidelines that quantify a daily limit for sedentary time^16^; however, the Canadian 24-hour movement guidelines suggest that OAs should spend no more than 8 hours per day being sedentary.^26^ Hence to improve the quality of life in OAs, it is vital to minimise sedentary patterns and enhance activity.

Epidemiological and meta-analytic data show that both low levels of moderate to vigorous physical activity (MVPA) combined with substantial amounts of SB are simultaneously related to significant poor health outcomes and mortality.^27^,^29^ The WHO PA and SB 2020 recommendations encourage individuals to reduce extended sitting time; however, no particular technique has been suggested to help people reduce their sitting time.^30^ Presently, a few studies have assessed the feasibility of SB interventions that specifically target reducing OAs’ SB. The majority of these interventions have primarily focused on individual consultations and feedback sessions on behaviour change, action planning and goal setting.^31^,^36^ Additionally, a pilot study on OAs integrated a habit formation approach, aiming to replace SB with light activity. They used self-monitoring sheets every week and a booklet with techniques for overcoming extended periods of SB by introducing active habits to replace typically SBs.^37^ Findings from these intervention studies showed good feasibility and retention rates in minimising SB, highlighting that the interventions could successfully reduce SB.^37^ Hence, behaviour modification strategies have proved promising for minimising sitting behaviours and increasing activity.^38 39^ Furthermore, Rosenberg *et al*^40^ carried out an intervention study using wrist-worn wearable activity trackers (WATs; electronic wearable technologies used to track PA and SB) to provide vibrations after every 15 min to cue breaks to sedentary time during the entire day along with health coaching sessions and written materials. The study findings showed that the multi-component interventions could be implemented, as they demonstrated feasibility and increased OAs’ level of satisfaction with the intervention. However, these previous intervention studies have included younger adults with a minimal age of 50 years^33 41^ or 60 years,^3132 34^,^37 40^ although according to definitions and guidelines these people would not be classified as ‘older adults’ as they are not ≥65 years.^42^,^44^ Therefore, there has been limited research on the effectiveness of interventions to decrease SB, particularly among OAs.^45 46^ This is a significant omission since younger adults may benefit from different behaviour change techniques (BCTs) and interventions than those that may be of benefit for OAs.^47 48^ Including younger adults in these interventions might have positively influenced the results because younger people may be more willing to change their behaviour and face fewer barriers to breaking up their SB. Therefore, interventions that were successful in reducing SB in younger persons might not be feasible, acceptable or practical for OAs,^49 50^ resulting in the need to investigate the use of these interventions with OAs. Additionally, there is a dearth of studies including participants from the multi-ethnic sedentary OA group.^51 52^ Based on the high chronic disease prevalence in this group, research in this population is extremely important^53 54^ as they would arguably benefit more from SB reduction interventions than other ethnic groups of OAs.^32^,^37^ However, prior to conducting an intervention trial, it is important to assess the feasibility of an intervention, in accordance with the Medical Research Council (MRC) recommendations.^55^ In light of this, the current study aims to examine the feasibility of a multi-component intervention aimed at decreasing SB and employing a wearable device, along with a brief health coaching session, a pamphlet and reminder messages, with OAs who are at least 65 years old and come from a multi-ethnic background.

## Methods

### Study design overview

This study will employ a multi-methods design, generating quantitative data and using subsequent semi-structured interviews to produce qualitative data on OAs’ experiences of the intervention and procedures. This feasibility study will be carried out and reported in accordance with the Standard Protocol Items: Recommendations for Interventional Trial guidelines to ensure appropriateness of the reporting of the protocol^56^ (online supplemental file 1). The study will recruit approximately 20–25 OAs and is planned to be conducted between 1 January 2024 and 31 May 2024. This range of sample size is not based on statistical power but was an appropriate and practical sample for a feasibility study^57^ considering resource (time, money and effort) constraints. This sample size is similar to that used in other feasibility studies in previous studies of non-pharmacologic interventions that explored similar outcomes and led to further effectiveness trials.^31 36 49 50^

### Study intervention

The intervention will be based on behavioural theories,^58^ including the socio-ecological model^59^ and habit formation model.^60^ The socio-ecological model emphasises the importance of taking into account factors that may have an impact on SBs at the level of the built environment, including the home and outdoor environment. According to the principles of habit formation, automatic and unconscious processes have a particular influence on decisions to sit. Standing (rather than sitting) decision-making will become more automatic with practise if it is first brought into conscious awareness.

During the 12-week study period, OAs will receive an individualised face-to-face brief health coaching session lasting 40–60 min, along with a pamphlet, WAT and reminder messages via a mobile phone. In addition, the researcher will be available to the OA participants via telephone or email should they have any additional questions, problems or concerns throughout the study.

The neck-worn activity tracker provided to the OAs was built using off-the-shelf components, an Arduino MKR Zero microcontroller board (with SD card slot and Li-Po battery support), an Adafruit LIS3DH tri-axis accelerometer, an Adafruit PAM8302 mono 2.5W Class D audio amplifier, a thin plastic speaker (8 ohm, 0.25W), a vibrating mini motor disc, a 2000 mAh 3.7V Li-Po battery and a Raspberry Pi 4 enclosure and is unavailable for commercial use. It also has a power on/off button and a micro-USB cable for charging via the Arduino board. The device is worn around the neck using a lanyard or a similar cord. The accelerometer is used to detect the orientation and change of movement of the device along all three (X/Y/Z) axes. The device can detect activity through its movement and can detect whether the person is sedentary (sitting or lying down). Although thigh-worn accelerometers are more accurate compared with other accelerometers (hip-worn or wrist or neck),^61^ we did not consider thigh-worn accelerometers in the present research as our previous experience suggests there are significant cultural rules in this participant group around body exposure and modesty that would make this difficult. Wearing a thigh-worn device might be considered as exposing or intrusive, which could make the OAs in our study feel uncomfortable. In addition, certain religious customs include dress restrictions that emphasise covering certain body parts. Wearing a device on the thigh could contravene these dress regulations, which would discourage participants from using them. A review has also highlighted that the attitudes of minority ethnic participants towards PA were highly influenced by religion and cultural dress codes.^62 63^ Hence, wearing an electronic device affixed to their thigh may cause discomfort and be considered culturally inappropriate among ethnically diverse OAs. We therefore chose to use a neck-worn device for these reasons because it is more convenient for users to put on and take off and, in general, is also preferred by OAs.^64 65^ Given that OAs prefer easy and user-friendly technology,^66 67^ neck-worn trackers are simple to use because they have fewer features than other trackers^68^ and hence have prolonged battery life, which minimises the need for regular recharging—which some OA users may find intrusive. Nevertheless, given the evidence that wrist-worn devices are popular due to their convenience and comfort, this study will explore if future iterations of the device need to be modified to enable them to be worn on the wrist.^69^

The device aims to provide alerts through vibrations and voice-based sound messages (such as ‘Move More—Sit Less’; ‘Thrive for activity within home space’; ‘Hustle for that muscle within the home’; ‘The less I sit, the less stiff I get’; ‘Tune your body into fitness’ and ‘Keep calm and move within the home’) after every hour of being sedentary within the home that will act as a prompt/cue to prevent prolonged SB. Participants will be required to walk or stand for a few minutes at the time of the interruption from the alert. Via these breaks in SB, OAs will be asked to increase their daily steps throughout the day. The purpose is to counteract SB using target behaviours: replacing prolonged sitting with any amount of stepping or standing to break up the time spent being sedentary. The OAs will not be given any other feedback from the device which is standalone, and OAs will be asked to switch it off using the power switch when it is not needed, for example, during water-related activities or when they are out of the home socially. It is possible that the lanyard could get caught onto something during a fall, thus to mitigate against this, a cord that is easily broken or detached will be used. In addition, in the reminder messages, OAs will be encouraged to use the device. The researcher will be available to the OAs via message, telephone or email should they have any problems related to the device or any concerns throughout the study. The researcher will also use this as an opportunity to encourage participants to wear the device during the intervention period.

The information booklet produced by White *et al*^37^ detailing SB and PA information in relation to OAs’ health and well-being will be used to design the health coaching session and pamphlets. This will be reviewed by the research team who are experts in Sport and Exercise Science and have extensive experience working with OAs. The intervention material will be available in English. The brief health coaching session will enhance participants’ awareness of SB and PA, knowledge of the detrimental independent effects of SB and inactivity on the risk of chronic disease development, and provide examples of ways of reducing SB. Here, it will be important that participants become aware that interrupting sedentary time and enhancing activity is important throughout the day.

The intervention pamphlet will outline the term PA, its benefits and the recommended level. It will also explain SB and the difference between being active and being sedentary, discuss the Canadian 24-hour movement guidelines and highlight ways of using the home for enhancing activity and minimising SB. This includes an everyday cue (eg, ‘when TV ads…’) and a performance behaviour when encountering the cue (‘…stand up or start walking within your home’).^37^ These examples will include less or more intense variations of the suggested activities or behaviours within the home to enhance the likelihood of carrying them out.

The intervention pamphlet makes use of specific BCTs from the BCT taxonomy.^70 71^ The pamphlet promotes the benefits of activity and the health risks of prolonged sitting (ie, BCT 5.1: ‘Information about health consequences’’) and recommends lowering SB by directly substituting PA for existing sitting time (BCT 8.2: ‘Behaviour substitution’) or by enabling PA as one of the ways to replace sitting (BCT 4.1: ‘Instruction on how to perform behaviour’; BCT 13.2: ‘Framing/reframing’). The pamphlet explains that ‘PA’ not only refers to cardiovascular activity but also includes muscle-strengthening exercises, stretching and balancing. Additionally, it explains the habit formation technique and suggests including PA in daily routines, offering advice on how habits develop (BCT 8.3: ‘Habit formation’). Direction on forming habits is given through examples of activities that can be carried out within the home space, which will assist participants in minimising SB and increasing PA, improving their health and well-being. The pamphlet includes various examples of using the home environment to integrate activity into a daily routine (BCT 6.1: ‘Behaviour demonstration’) which, if practised regularly, will lead to a change in PA and SB habits (BCT 8.4: ‘Habit reversal’). These examples and reminder messages will act as cues to continue routine activity within the home environment (BCT 7.1: ‘Prompts/cues’) to reinforce and bring about favourable change in behaviour (BCT10.1: ‘Positive Reinforcement’). OAs will be asked to place the pamphlet in a convenient place that will remind them to use it frequently and make consulting it part of their daily behaviours.

The majority of behaviour modification programmes have transient benefits that dissipate when the intervention ends.^72^ This may indicate a gradual loss of motivation or external support.^73^ A sustainable intervention that reduces sedentary patterns and increases activity is therefore much needed. According to behavioural psychology, developing ‘habits’ can secure improvements in behaviour over time. Habitual behaviours are defined as activities motivated by impulses that are triggered by confronting a situation (environmental cue) in which the behaviour has been carried out on a regular basis in the past.^74^ Examples of environmental cues might be a person, location or prior activity within an established routine.^75^,^77^ A mental cue-behaviour association is strengthened by context-consistent repetition^78^ that triggers an impulse to perform necessary actions when an individual encounters a cue.^74^ Efforts and encouragement are needed to begin and maintain the early phases of the formation of habits; as the habit is formulated, control of behaviour is transferred to the external cue, and the activity requires less conscious effort to complete.^79^ As long as the cue is encountered, habitual behaviours are predicted to continue,^80 81^ possibly leading to self-perpetuating behaviour. Thus, habit development presents a viable strategy for sustaining the benefits of behavioural interventions following the termination of external support.^80^ The planned multi-component strategies will focus on how to alert oneself to have regular breaks from sitting which will help OAs use strategies to modify their inactive behaviour during the entire day.

### Eligibility criteria and recruitment

Different community organisations working with multi-ethnic OAs (eg, Asian community, African community) in Swansea will be contacted to request permission to recruit participants through their contacts and members. Local coordinators and sub-coordinators will be approached as they serve as gatekeepers to the recruitment of these OAs. Additionally, recruitment will be done via the sport, play and community networks of the city and county of Swansea, as well as advertising throughout the university where the researchers are located, using the intranet. Recruitment posters/leaflets, letters and email will be used for advertising the study.

Older individuals who are interested will be asked to contact the area project coordinator in their region to express their interest. The researcher will then contact interested participants. Those who are willing to participate will receive a study information sheet. The lead researcher will schedule a mutually agreeable time to meet with them and discuss the study process and procedures with them. If the OA is satisfied to proceed, they will be provided with a consent form to take part in the study.

We will employ a non-consecutive eligibility approach to recruit 20–25 participants from community organisations, which has been identified as a large enough sample to assess the feasibility of the intervention.^3157 82^,^85^ Eligible OAs aged ≥65 years, either male or female, sedentary (self-reported ≥5 total leisure hours sitting per day) and able to communicate in English or Urdu will be included. OAs with physical impairments that hinder them from participating in daily light-intensity PA, those who are currently engaged in daily MVPA (>150 minutes/week) or those who have participated in the past 3 months in a study promoting PA or reducing SB will be excluded. OAs with mental impairment, who are unable to provide informed consent or are unable to cooperate with the research team for the full duration of the project will not be included.

### Data collection

Pre-intervention and post-intervention measures of PA, SB, self-efficacy, self-report habits and well-being will be completed by participants. Socio-demographic characteristics of OAs will be measured at baseline. A qualitative interview will also be conducted using a semi-structured interview guide after the post-assessment period to assess the acceptability of the intervention. Interviews will be conducted in Urdu or English, with those that are conducted in Urdu translated into English by the primary researcher who will conduct these interviews.

PA will be measured with a wrist-worn accelerometer (ActiGraph wGT9X Link+, Pensacola, Florida, USA). The ActiGraph is reliable^86 87^ and valid for use with OAs,^88 89^ people with mobility limitations^89^ and obese adults.^90^,^92^ To measure each OA’s PA, they will wear the device on their non-dominant wrist for 7 days during waking hours and will remove it during any water-related activities such as bathing or swimming.^93^,^95^

OAs will be given a log sheet and asked to keep track of the times the accelerometer was worn and was removed throughout the day, describing the timings and reasons for each removal.^94 96 97^ Moreover, each OA will receive instructions on how to attach and wear the device for 7 consecutive days. They will also be asked to maintain a sleep log, recording hours slept and waking and rising times. This approach is beneficial to differentiate between wear and non-wear time and it also encourages compliance by acting as a reminder.^98^,^100^ Accelerometer data will be processed using ActiLife V.6.8.0 with data captured in 60 s epochs with the low-frequency extension enabled. The downloaded data will be subjected to compliance and quality control checks, and log sheets will be assessed to check the accuracy of wear time algorithms and to remove the non-wear time from the study.^96^

Total SB will be self-reported using the Measure of Older Adults’ Sedentary Time (MOST),^101^ which has been validated via objectively assessed sedentary time. It is divided into seven domains: reading, using a computer, watching television, socialising with friends and family, time on transport vehicles, doing hobbies or other activities. MOST data will be summed across the seven activities to provide a combined score. While exact minimal clinically important difference (MCID) values for MOST are unavailable, there are relevant studies and reviews that offer insight into what constitutes a meaningful change in SB. These identify that a reduction of 30–60 min can be seen as a significant difference.^88102^,^104^. Self-efficacy for exercise will be measured using the Self-Efficacy for Exercise (SEE).^105^ This tool is used to measure self-efficacy for activity. The SEE is a self-report measure with five items assessing one’s degree of confidence in the ability to perform the activity. The score is the sum of the item responses; higher scores indicate greater self-efficacy. There is no established MCID for SEE^106^; however, for the present study, the Minimal Important Difference (MID) will be estimated at around 1 to 2 points on the scale.^107^ The Self-Report Habit Index (SRHI)^37 108^ will be used to measure habits associated with SB (ie, sitting) and PA, which has been validated in previous studies.^109 110^ The MCID for the SRHI is not as widely established as for some other scales. However, research suggests that a change of about 0.5 to 1 point on the SRHI scale represents an MID. The Warwick-Edinburgh Mental Well-being Scale,^111^ a 14-item self-report measure, will be used to assess well-being. Respondents rate their experience regarding each statement over the last 2 weeks. Each item is scored using a 5-point Likert scale ranging from 1 (none of the time) to 5 (all of the time), with the total score ranging from 14 (low well-being) to 70, and the MCID is represented by a change of three points.^112 113^

### Feasibility assessment

The primary outcome is the acceptability and feasibility of the procedures of the intervention and recruitment and retention measurement. Recruitment rates were determined as the ratio of study participants who consented to participate out of those invited and eligible to participate. We will assess the attendance at, and time taken (minutes) for, the face-to-face health coaching session. Similarly, we will note the number of sent and successfully delivered messages and record the number of responses to, and acknowledgements of, the messages. Further, weekly reading/usage of the pamphlet will be recorded. We will use qualitative interviews to determine their overall user experience with the intervention which will help us to assess device feasibility by examining their perception regarding the vibrations and sound messages emitted from the device.

In addition, we will record drop-out reasons for discontinuing study participants, as well as adverse events experienced (eg, hospitalisation, life-threatening events, death). The suitability of measurement procedures will be evaluated based on the completion rates of the measures.

### Semi-structured interviews

Following the completion of the intervention, semi-structured interviews will be conducted with enrolled OAs who participated in the trial to understand the intervention’s feasibility and acceptability. The interview guide is based on the UK MRC framework. A key component in planning health-associated interventions is the feasibility phase, which produces important information on areas such as acceptability, usability and functionality that will be assessed using the interview guide (online supplemental file 2).

Interviews will be held either face-to-face or via telephone, Zoom or any other appropriate interface to which the participants have access and will be conducted by the primary researcher (NAAM). Enrolled OAs will be interviewed to explore information about the participants’ overall experiences (acceptability, usability and functionality) of using the multi-component intervention and its impact on their PA and SB, any problems they encountered during the intervention and how they feel it could be improved. All interviews are expected to last between 40 and 60 min and will be audio-recorded to allow subsequent verbatim transcription. If interviews are conducted in Urdu, they will be translated into English by the primary researcher (NAAM). The reporting of the study will be in accordance with the COREQ checklist to ensure transparent and comprehensive reporting of qualitative research. The checklist is appended in online supplemental file 3, detailing specific items related to the research team, study design and data analysis.

### Data analysis

The primary researcher is accountable for the initial data entry. Statistical analyses will be done in SPSS, V.17.0. The intervention feasibility will be assessed through uptake, adherence and data completeness. Changes from baseline to post-test data in sedentary time, PA, well-being, self-efficacy and self-reported habits will be measured using paired t-tests. Linear regression analysis will be used to assess the impact of socio-demographic (age, gender, ethnicity and socio-economic status) and home characteristics on PA and SB, with the significance level set at p value <0.05.

Qualitative data analysis of interview data will use a thematic analysis technique to identify the common themes regarding the feasibility of the trial. The first step will use a combination of both inductive and deductive analysis using an open coding technique to identify themes and codes that give insights regarding the feasibility of the intervention and a future effectiveness trial. Initial deductive codes will be determined by the MRC framework. Two researchers will analyse the data independently; they will then have a detailed conversation about the themes and subthemes until an agreement is reached. In the next step, the identified themes and subthemes will be interpreted using the study’s underpinning models and theories to enable deeper interpretation and explanation of the study findings. The quantitative and qualitative data will then be combined for interpretation and reporting. The integration of the data will provide knowledge about the feasibility of the intervention which will also assist us in guiding an effective trial in the future.

### Study timeline

The intent is to initiate the feasibility trial by 1 January 2024. We estimate that it will take 3–4 months to recruit 20–25 OAs and the study will be completed by 31 May 2024. By the beginning of September 2024, it is anticipated that the main analysis will be finished.

### Patient and public involvement

There is no patient or public involvement in setting the research agenda.

## Ethics and dissemination

Ethical approval for this study has been obtained from Swansea University Research Ethics Committee (NM_31-03-22). Informed consent will be obtained from participants. The information shared by the participants will remain private and confidential. A unique ID will be given to all participants. No reporting of the results will include the names of the participants, and participants’ data will not be connected to personal information. Only the research team will have access to the collected data. Participants’ quotes that will be used in reports and publications will remain anonymous. The findings of the study will be disseminated to the scientific community through conference presentations and scientific publications.

## Discussion

Carrying out research with OAs has several challenges,^114 115^ particularly when involving multi-ethnic OAs as in this study, in part contributing to their under-representation in research.^51 52^ Given the high prevalence of chronic disease among multi-ethnic minority OAs, research attempting to understand and modify their activity and sedentary patterns is important.^53 54^ It might be that ethnically diverse populations have distinct and unique perspectives on the medical, academic and research community at large, as well as the particular research institute carrying out the study. As a result, social and cultural aspects that influence the recruitment and retention of OAs from under-represented communities require further consideration.^52^ The recruitment of multi-ethnic OAs in research studies is a challenge that needs careful and rigorous planning to determine the target population to plan effectively for personnel/staff, budget, research procedure and resources to enrol and retain participants. Additionally, planning and maintaining a long-lasting, inclusive, bi-directional relationship with a suitable group prior to research recruitment is a productive way to gain insight and direction as well as to establish trust.^52^

The current research will include multi-ethnic OAs (≥65 years), which is important in enabling generalisation of the findings to this age and ethnicity group. The utilisation of ActiGraph before and after the intervention will permit the researcher to objectively examine PA. However, during the intervention, there might be a concealed effect due to using self-reported SB data, the authenticity of which has been questioned.^116^ Nonetheless, assessing self-reported SB provides beneficial data as it allows researchers to find out more regarding the specific types of sedentary activities undertaken (such as watching TV, reading and socialising) that are relatively unexamined and contribute to the total sedentary time.^117^ This information is impossible to collect with an objective activity device but may have an important influence on a range of outcomes related to physical and mental health^117^ and will help in designing an intervention that targets specific SBs to account for this. Future trials might therefore focus on both subjective and objective measurement to assess SB in OAs, in addition to including a control group and follow-up measures of long-term intervention adherence. In the current study, it will not be possible to draw conclusions about the intervention’s long-term impacts, and removing the prompts at the study end might lead participants to return to their unhealthy sedentary patterns.^118 119^ It is expected that individuals may still need the cues to reduce their SB even after the intervention has concluded as behaviour modification requires time.^120^ Following this study, the effective intervention trial, using objective and subjective measures of behaviour, a control group and long-term follow-up measures, will enable us to examine if behaviour changes can be sustained. In addition, in the current study, the researcher who will deliver the intervention will also carry out the interviews; thus, results may be biased by this dual role. However, the researcher will undergo qualitative training and keep a reflective diary to minimise her biases.

In summary, it is anticipated that the intervention will provide a feasible, convenient and practical way to enhance OAs' activity and minimise SB; hence, the study findings may have important implications for public health. With the increase in the population of OAs, it is critical to provide practical strategies to help all OAs remain independent and autonomous in later life.

## supplementary material

10.1136/bmjopen-2024-084645 online supplemental file 1

10.1136/bmjopen-2024-084645 online supplemental file 2

10.1136/bmjopen-2024-084645 online supplemental file 3
