# Supplementary material for: A multi-method feasibility trial of a multi-component behaviour change intervention to reduce sedentary behaviour and increase physical activity among ethnically diverse older adults
Source: BMJ Open. 2024 Nov 7;14(11):e084645. doi: 10.1136/bmjopen-2024-084645 (PMC11552559; doi:10.1136/bmjopen-2024-084645)
Supplement: online supplemental file 2 [file bmjopen-14-11-s002.pdf]

In-Depth Interviews

Acceptability

1. What has your experience with using multi-component intervention (pamphlet, wearable device and health coaching session and reminder messages)? What part of the interventions were helpful/useful for you that you liked?
2. b) What features/ components do you find least helpful or useful? – or you like least?
3. What are the top 2 or 3 most important or helpful parts of intervention that we cannot do without?
4. Are there any features of the intervention that you find difficult to use?
5. What do you think about the intervention duration of 3 weeks? Was it too long or short?
6. To what degree does the intervention program meet your expectations?
7. Have you had any problems with the equipment functioning? If yes

a) What did you do to resolve it? Or was the problem easily solved?

8. Were there any factors that positively or negatively influenced intervention use? (pain, stiffness, workload, stress, family)

Demand

9. What did you think of the changes in your sedentary time from participating in the program?
10. Has the intervention program helped you to take more breaks from sitting? If yes, what helped you? If no, what made it hard to?
11. Has the intervention program helped you to add more periods of standing time into your day? If yes, what helped you? If no, what made it hard to?
12. How often do you consciously (take break from sitting)?
13. How did your perceptions or attitudes towards your own sedentary time change while you were in the study?
14. How did the intervention program fit/not fit into your daily life? Or please explain how you use the intervention program within your daily **life/PA?**
15. **How did the program impact your physical activity?**
16. Which element of intervention helps you to increase activity and break your sitting time?
17. How did intervention help you to make changes to your home environment to do more PA and lessen SB?

Implementation:
Health Coaching Sessions:

- What are your thoughts on your experiences regarding your health coach sessions? Satisfied
- What did you think about the number of sessions (was single session was enough)?
- What did you think about the content? Would you have preferred another type of content?
- What did you really like/dislike about the health coach sessions?
- What could be done to make the sessions more effective?
- How would you have engaged in the program if we gave you an online health coach session?

Pamphlet

- What are your thoughts regarding the pamphlet?
- What did you think about the content? Would you have preferred another type of content?
- What did you really like/dislike about the pamphlet?
- What could be done to make the pamphlet more effective?

Reminder messages:

- Did you find reminder messages helpful?
- Did they encourage you to do more activity and minimize SB?
- Did the messages were timely and easy to understand?

Health benefits:

Did you notice any changes in your health and well-being as a result from participating in this study (physical or mental)?

- How did you feel on days you sat less?
- What changes have you noticed in your body? (Prompt: stiffness, pain, fatigue or ability to do daily activities)
- What changes, if any, have you noticed in your mood? (Prompt: depression, anxiety, cognition, ability to focus, energy level, etc.)

Wearable tracker device

Did you ever receive automated reminders to break your sedentary behaviour?

) What do you think of these? Were they helpful or not?
) What do you like/not like about the these automated device?
) Do you like the reminders or find them annoying?
) Was it easy to use the device?
) Has the reminder caused you to break sitting time and add more periods of standing?
) What modifications do you want in the automated reminders via wearable device?
) What other technologies would have been helpful to have?

Actigraph:

a) Was it easy to use the wrist-based activity monitoring devices before and after the intervention for 7 consecutive days? Or do you face any challenges?
b) Do you find it difficult to log your wake up and sleep time and non-wearing time?
c) Do you find it difficult to fill out the questionnaire?

Practicality

2. How would you feel if, to use the activity device, you had to use your own personal Smartphone?
a) Do you expect it to be easier or more difficult to use? And why?
b) Would this change the way you use the activity device or incorporate it into your daily activities?
c) Do you think you would have required different instruction and guidance?

d) Would this change your willingness to use the activity device?
e) Could you think of other ways, besides using intervention, that would help you to increase physical activity and minimize sedentary behavior? If yes, what are they?

Limited Efficacy
1. How would you find your relationship with research staff?
a) Do you feel that they support or help you in intervention program?
b) What is your opinion of the instruction you received?

Family/Friends:
1.What do your family and friends think about you being part of the intervention program?
a) Are they encouraging?
b) Do they see the value it in?

Recommendations:

1.What would encourage OA to take part in study?
a) Would you recommend intervention programs to other older adults?
b) If you were asked, would you take part in a larger trial using the intervention? If no, why not? (here you can probe which parts of the intervention would deter them from being part of a larger/longer study).

Conclusion

- What do you think will happen now that the study has concluded? What strategies do you plan on continuing to use?
- Is there any other feedback you would like us to have that would make the intervention program better?
- Are there any other important issues or anything you would like to add that we haven't discussed?
- Overall satisfaction level?

Thank you so much for allowing me to come and interview you. Your time and your views are greatly appreciated. All the information you've provided will be very helpful in shaping future research, policy and programs in the area of older adults' activity, housing and health.
