# Supplementary material for: A multi-method feasibility trial of a multi-component behaviour change intervention to reduce sedentary behaviour and increase physical activity among ethnically diverse older adults
Source: BMJ Open. 2024 Nov 7;14(11):e084645. doi: 10.1136/bmjopen-2024-084645 (PMC11552559; doi:10.1136/bmjopen-2024-084645)
Supplement: Supplementary file 4 [file bmjopen-2024-084645.reviewer_comments.pdf]

PEER REVIEW HISTORY

BMJ Open publishes all reviews undertaken for accepted manuscripts. Reviewers are asked to complete a checklist review form and are provided with free text boxes to elaborate on their assessment. These free text comments are reproduced below.

ARTICLE DETAILS

Title (Provisional)

A multi-method feasibility trial of a multi-component behaviour change intervention to reduce sedentary behaviour and increase physical activity among ethnically diverse older adults.

Authors

Meghani (NAAM), Naureen Akber Ali; Hudson (JH), Joanne; Stratton (GS), Gareth; Mullins, Jane; Sahoo (DS), Deepak

---

VERSION 1 - REVIEW

**Reviewer**	**1**
**Name**	**Baldwin, Claire**
**Affiliation**	**Flinders University College of Nursing and Health Sciences**
**Date**	**13-Mar-2024**

COI **None specific to declare - other than I am also currently conducting a similar feasibility study (multi-methods) of a health coaching and support type intervention in older adults but from hospital to home. So a number of similarities with the current registered trial in terms of the intervention approach, methodology and outcome measurement domains.**

Well done on a clearly defined and reported study protocol, that addresses an important need for intervention development in the field of PA and SB research for older adults.

Review title

Suggest revision of the title to include

- Mention of the methods design as well as feasibility, which is perhaps more of a multi-methods study than mixed method – unless there is more detail in the analysis about how/what integration will be done

- Better description of the intervention - I'm not sure that the intervention description in the title best reflects what the core components are and what is being done. Optimising 'home space' to me sounds like an intervention about environmental adaptations or equipment to support older adults activity within that setting. The presented intervention is more of a

multi-component/strategy behaviour change or coaching intervention, so suggest revise that to something that the authors consider more aligned

Introduction

The introduction is well written and explains the background to the concepts of PA, SB and the problems in a general community dwelling population of older adults. The intervention description similarly well describes in detail and justifies the use of behaviour change theory and components that should mean this intervention has a reasonable likelihood of effectiveness.

- If there is word count and capacity, I do think that some coverage of what other similar interventions there are and their results have been done using this approach of coaching, device wearing for feedback and information. It sounds like it has similarities to https://pubmed.ncbi.nlm.nih.gov/32498040/ which you reference the protocol for but could update to the published version, and other coaching based programs https://www.comebacktrial.org.au/ In essence it would be helpful to have a clearer outline of 'at this point in time…. what has already been learned/done in these types of health coaching interventions' to understand where your study fits in with the desired next step plans of a larger trial. It may not need to be included in the protocol reporting, but have you also done any searches of trial registers to see if there are any other similar studies in progress?

Study intervention description

Mention is made of a neck worn activity tracker (page 6 line 59), but with very other little detail. It reads like this is part of the intervention (and intervention tool) rather than part of outcome measurement – is that correct? Either way, I would like to see a more detailed explanation of what this device is, including

- Is this a commercially available device or not (suspect not), but what details of manufacture etc?

- What is its validity of detecting sedentary behaviour and in this wear location (as usually this would be done with thigh worn type devices or similar that can detect differences between sitting and standing, or like e.g. Garmins/Fit bits where its wrist worn and an acceleration based algorithm)

- Are there any known issues of wearability – e.g. do people like wearing something around their neck rather than another wear location (like thigh, wrist, waist) and are there any issues with safety and something around the neck for older adults (e.g. don't want this to get caught if they have fall?). Or any anticipated issues with the alerts (e.g. how do they silence the voice alerts if they are out socially and don't want them at that time, or to adjust for sleep/bed times)

- How will they be encouraged or monitored to use this tracker, is it intended to be every day for the 12 weeks?

Eligibility criteria and recruitment

Page 8 line 14 – states 'diverse communities' will be contacted. Can the writing please be more specific about what types of diversity or what is meant by diversity? E.g. within the older adults age bracket, or different cultural and/or linguistic diversity or different physical or cognitive capabilities?

Sample size

Page 8 line 31 – if these are to be consecutively eligible recruited patients, please state so.

Page 8 line 34 – if translated intervention materials are required (or interview transcripts), please indicate how this will be done.

Data collection

Page 9 line 7 – optional feedback to consider about processing of Actigraph data – as I don't think there is a clear answer or precedent for researchers in managing this data in this population. But, i'd caution and re-visit if you want the low frequency extension (LFE) filter enabled or not – if already started then probably don't change it, but think about this especially being a community dwelling older adult population. In my experience, I have found it difficult to determine cut points for analysis of the actigraph data in this age group with the LFE on and thinking about how active/inactive this group is. So you can still use it but would suggest a re-check of literature and how you will then process and present the data in your analysis plan, e.g. using sedentary, light, MVPA thresholds, steps or METs or something else.

Page 9, line 13 - Given a main goal of the study is to detect changes in SB, its unfortunate that this is only being measured by self report and the MOST (event though it is a good measure) and not a more objective device derived measure, as the changes in SB may not be very marked or sensitive to be picked up (e..g what if you only get a shift of 10, 20 or 30 min SB time per day? What would you see as a minimal clinically important difference in SB for your study participants from pre to post?). Still given this is a feasibility study, this could be acknowledged as a limitation, but if its to be a primary outcome in a future trial it would be worth thinking about and planning for what your primary measures in that would be (so you could do a sample size calculation).

Page 9, line 13 - Are you able to prospectively cite what you interpret and understand a MCID/MID to be on all of these different questionnaire/scale based outcome measures? So its clear at the outset what a meaningful change score would be from pre-post intervention?

Page 9, line 45 – adherence assessment. As currently expressed, is this adherence outcome more about (qualitative) data completeness than adherence to the intervention components? Id be more interested for adherence in things like the actual duration (and ? content) of the initial health coaching session, or the wear of the neck device or the number of SMS follow ups and subsequent phone calls or emails and how they were spaced out over

the 12 week intervention. In other words, so you get some more details about intervention fidelity.

Semi structured interviews

Page 9 line 51 - I can see a COREQ checklist (completed to the relevant stage for a protocol) has been included as an attachment but is not referenced within text here as an appendix/supplement.

- I wonder if rather than having the details in the checklist, you include some/all the relevant detail in the methods text here and indicate that the COREQ checklist will be used for the full study reporting? Otherwise, make sure that references in text are made to any of the supplementary materials.

- Reference to the COREQ checklist also prompts me to also wonder what other reporting guideline you intend to use for the other observational aspects of this pre-post (single group) study?

- Also, do you have any more information on the development of the interview guide (e.g. was a theory or framework used to identify key question themes) and any pilot testing?

Patient and public involvement

Page 10, line 36 – this sentence is not worded as clear as it could be. How will older adults be informed through the data collection interviews (doesn't seem to make sense - other parts ok). Do you mean involved as study participants in the intervention and outcome assessments (that include interviews and other measures)? How are they involved in the protocol or dissemination of data?

Discussion

Page 10 line 29 - Are there any potential limitations to acknowledge at the outset in this discussion section (aside from the limitations section elsewhere in the manuscript)? Or suggest already what future directions may be, e.g. how do you want to use the quantitative data to inform a future trial design?

Strengths and limitations section.

States 'It is anticipated that the older adults enrolled in this study will find it challenging to wear the device consecutively for seven days. However detailed guidance and instruction will be provided that might ease this challenge.' Can you please outline what device is meant here, the actigraph or the neck worn device? If mentioning an anticipated challenge with a device in this summary section, I would like to see a clearer link/mention elsewhere in the paper about where this hypothesis/limitation is going to be tested (e.g. is it a question in the interview guide, or use of wear logs for devices), otherwise consider if there is a different key point that is better to highlight about either your study methodology (e.g. single group and no control group (which is probably ok for the stated study feasibility aims) or no longer

term follow up beyond 12 weeks about potential sustainability of behaviours) or the intervention itself, in this limitations section.

**Reviewer**	**2**
**Name**	**Rodrigues, Isabel**
**Affiliation**	**McMaster University, Rehabilitation Sciences**
**Date**	**28-May-2024**
**COI**	**None**

Dear Naureen and co-authors,

Thank you for the opportunity to review your protocol. The protocol is overall well written and the ideas are clearly articulated. I also commend the authors for focusing on an issue that does not get a lot of research (sedentary behaviour interventions). As a researcher who also does a lot of sedentary behaviour and knowledge translation research I have some considerations to improve the protocol:

1. The main objective of the study is to "examine the feasibility of a wearable gadget to remind users to break up their sitting time". Some of the outcomes you have selected to assess feasibility (i.e., uptake, data completeness) don't necessarily address your main objective. The purpose of a feasibility study is to answer the question "can this study be done"? There has been several studies on sedentary behaviour where older adults used a wearable device to assess sedentary behaviour - so it's unclear to me why a feasibility study is needed. Is there something specific about this device that requires an additional feasibility study? I wasn't sure if this was actually supposed to be a pilot study (a smaller version of the main study used to test whether the components of the main study can all work together). There is some really good work and guidelines that were published by Dr. Lehana Thabane on feasibility and pilot studies that I recommend you review to help parse out why a feasibility study is needed. Dr. Thabane's paper also goes into the different types of feasibility outcomes depending on your research question (e.g., feasibility resource, feasibility process).

1b. In my own studies, I found certain groups have a lot of mistrust and distrust when using tracking devices. I am wondering how that will be captured in the study? Is there a questionnaire or an exit interview that will take place to capture why they dropped out?

2. I am unsure about how you will identify individuals that are sedentary. According to the 24-hour Canadian guidelines, someone who is sedentary would be an individual that engages in sedentary behaviour for >8 hours per day, has >3 hours of screen time, and does not break up their sedentary behaviour often. You are including individuals who have a self-reported sitting time ≥5 hours; however, this does not mean they are sedentary. A deeper

explanation on how you selected this 5 hour cut-off would be helpful. The other problem to consider is that several individuals may accumulate several hours of sedentary behaviour, but don't consider themselves sedentary because it's "active" or "purposeful".

3. There is some evidence that seasonal differences affect how sedentary individuals are, with older adults being less sedentary during the warmer months and more sedentary during the colder months. I am wondering if you have considered this variation in sedentary behaviour in your inclusion criteria and in your intervention consideration your intervention takes place in winter and early spring.

4. I noticed gender has not been considered in the protocol. Men and women engage in different sedentary behaviours due to gender norms and roles. How has that been considered in the study?

5. In terms of the intervention, I am having difficulty understanding why this intervention is different from some previous interventions that have used the same behaviour change techniques. I think one thing to consider is that there is new research that shows most older adults who are healthy are accumulating over 8 hours of sedentary behaviour, but they are also getting up every hour to 1.5 hours. Technically they are breaking up their sedentary behaviour. The second thing that research has shown is that some sedentary behaviours are valued for resting, socializing, and daily functioning, and should not be targeting. Thus not all sedentary behaviours should be targetted, and interventions should move away from targeting total sedentary time to decreasing sedentary behaviours that are not meaningful or have negative health consequences (e.g., consider watching TV versus reading a book for 1 hour). There was a Cochrane review recently published by Chastin that showed none of the behaviour change techniques to target sedentary behavior in older adults were effective - probably because context of sedentary behaviour was never considered (i.e., targeting total sedentary times rather than those behaviours that are bad for health).

6. Could you please provide more detail about how the pamphlets and other components were designed? Were they designed and reviewed by patient partners? What was the process used to design them? What evidence was used to design them?

7. Why are wrist worn accelerometers used instead of thigh worn? The accuracy of the wrist worn is a lot lower than the thigh worn accelerometers. There was also a paper that was recently published that suggested using two accelerometers (e.g., thigh and ankle) to improve the accuracy of detecting sit-to-stand movements

8. I also recommend collecting medical history, and other outcomes listed in the CIHR equity lens PROGRESS-PLUS.

9. You mention that you will used reflexive thematic analysis, but the methods don't align with Braun and Clarke's methods of reflexive thematic analysis. I would include a section on your epistemology, positionality, how data credibility will be determined, audit trail, etc. The

way the methods are currently written comes across as a thematic context analysis instead of a reflexive thematic analysis.

10. The discussion could use more work. I would expand on how this intervention is different from other interventions and why the components you've included may be effective and sustainable. I would also include a paragraph about what you predict will happen in terms of the feasibility and limitations of the study and how you plan on overcoming these limitations.

11. It's not clear to me how the sample size was selected or calculated. A description of why 20 to 25 older adults is needed would be helpful.

VERSION 1 - AUTHOR RESPONSE

**Reviewer 1 Comments to Author:**	**Responses**:
Dr. Claire Baldwin, Flinders University College of Nursing and Health Sciences<br>Comments to the Author:<br>Well done on a clearly defined and reported study protocol, that addresses an important need for intervention development in the field of PA and SB research for older adults.	Thank you so much for sharing your expertise with us; we appreciate the time you have taken to scrutinise and improve our protocol.
Review title<br>Suggest revision of the title to include<br>- Mention of the methods design as well as feasibility, which is perhaps more of a multi-methods study than mixed method – unless there is more detail in the analysis about how/what integration will be done<br>- Better description of the intervention - I'm not sure that the intervention description in the title best reflects what the core components are and what is being done. Optimising 'home space' to me sounds like an intervention about environmental adaptations or equipment to support older adults activity within that setting. The presented intervention is more of a multi-component/strategy behaviour change or coaching intervention, so suggest revise that to something that the authors consider more aligned	Thank you so much for your feedback. We have revised the title. Changes are done on page 1, line number 3-4.<br><br>Revision of the title:<br>A multi-method feasibility trial of a multi-component behaviour change intervention to reduce sedentary behaviour and increase physical activity among ethnically diverse older adults.
Introduction<br>The introduction is well written and explains the background to the concepts of PA, SB and the problems in a general community dwelling population of older adults. The intervention description similarly well describes in detail and justifies the use of behaviour change theory and components that should mean this intervention has a reasonable likelihood of effectiveness.<br>- If there is word count and capacity, I do think that some coverage of what other similar interventions there are	Thank you so much for your feedback. We have revised the introduction. The suggested reference has also been added. Changes are done on page 5, line number 3-34 and page 6, line no. 1-2.

and their results have been done using this approach of coaching, device wearing for feedback and information. It sounds like it has similarities to https://pubmed.ncbi.nlm.nih.gov/32498040/ which you reference the protocol for but could update to the published version, and other coaching based programs https://www.comebacktrial.org.au/ In essence it would be helpful to have a clearer outline of 'at this point in time…. what has already been learned/done in these types of health coaching interventions' to understand where your study fits in with the desired next step plans of a larger trial. It may not need to be included in the protocol reporting, but have you also done any searches of trial registers to see if there are any other similar studies in progress?	
Study intervention description<br>Mention is made of a neck worn activity tracker (page 6 line 59), but with very other little detail. It reads like this is part of the intervention (and intervention tool) rather than part of outcome measurement – is that correct? Either way, I would like to see a more detailed explanation of what this device is, including<br>- Is this a commercially available device or not (suspect not), but what details of manufacture etc?<br>- What is its validity of detecting sedentary behaviour and in this wear location (as usually this would be done with thigh worn type devices or similar that can detect differences between sitting and standing, or like e.g. Garmins/Fit bits where its wrist worn and an acceleration based algorithm)<br>- Are there any known issues of wearability – e.g. do people like wearing something around their neck rather than another wear location (like thigh, wrist, waist) and are there any issues with safety and something around the neck for older adults (e.g. don't want this to get caught if they have fall?). Or any anticipated issues with the alerts (e.g. how do they silence the voice alerts if they are out socially and don't want them at that time, or to adjust for sleep/bed times)<br>- How will they be encouraged or monitored to use this tracker, is it intended to be every day for the 12 weeks?	Thank you so much for your feedback. We have revised the introduction. We have added all details. Changes are done on page 6, line number 30-32, page 7, line no. 1-34 and on page 8, line 1-9.
Eligibility criteria and recruitment<br>Page 8 line 14 – states 'diverse communities' will be contacted. Can the writing please be more specific about what types of diversity or what is meant by diversity? E.g. within the older adults age bracket, or different cultural and/or linguistic diversity or different physical or cognitive capabilities?	Thank you so much for your feedback. We have revised the sentence. Changes are done on page no. 9, line no. 23-24.

Sample size<br>Page 8 line 31 – if these are to be consecutively eligible recruited patients, please state so.<br>Page 8 line 34 – if translated intervention materials are required (or interview transcripts), please indicate how this will be done.	Thank you so much for your feedback. We have revised the sentence. Changes are done on page no. 10, line no. 3-4.<br><br>Intervention material is used in English language.<br>Changes are done on page 8, line no. 6-9<br>Changes are done on page 12, line no. 17-18
Data collection<br>Page 9 line 7 – optional feedback to consider about processing of Actigraph data – as I don't think there is a clear answer or precedent for researchers in managing this data in this population. But, i'd caution and re-visit if you want the low frequency extension (LFE) filter enabled or not – if already started then probably don't change it, but think about this especially being a community dwelling older adult population. In my experience, I have found it difficult to determine cut points for analysis of the actigraph data in this age group with the LFE on and thinking about how active/inactive this group is. So you can still use it but would suggest a re-check of literature and how you will then process and present the data in your analysis plan, e.g. using sedentary, light, MVPA thresholds, steps or METs or something else.	Thank you so much for your feedback. We will consider it for our larger trial.
Page 9, line 13 - Given a main goal of the study is to detect changes in SB, its unfortunate that this is only being measured by self report and the MOST (event though it is a good measure) and not a more objective device derived measure, as the changes in SB may not be very marked or sensitive to be picked up (e..g what if you only get a shift of 10, 20 or 30 min SB time per day? What would you see as a minimal clinically important difference in SB for your study participants from pre to post?). Still given this is a feasibility study, this could be acknowledged as a limitation, but if its to be a primary outcome in a future trial it would be worth thinking about and planning for what your primary measures in that would be (so you could do a sample size calculation).	Thank you so much for your feedback. We have added important details. Changes are done on page no. 11, line no. 7-9.
Page 9, line 13 - Are you able to prospectively cite what you interpret and understand a MCID/MID to be on all of these different questionnaire/scale based outcome measures? So its clear at the outset what a meaningful change score would be from pre-post intervention?	Thank you so much for your feedback. We have added important details. Changes are done on page no. 11, line no. 13-23.
Page 9, line 45 – adherence assessment. As currently expressed, is this adherence outcome more about (qualitative) data completeness than adherence to the intervention components? Id be more interested for adherence in things like the actual duration (and ?	Thank you so much for your feedback. We have added relevant details. Changes are done on page no. 11, line no. 25-32 and on page 12, line no. 1-3.

content) of the initial health coaching session, or the wear of the neck device or the number of SMS follow ups and subsequent phone calls or emails and how they were spaced out over the 12 week intervention. In other words, so you get some more details about intervention fidelity.	
Semi structured interviews<br>Page 9 line 51 - I can see a COREQ checklist (completed to the relevant stage for a protocol) has been included as an attachment but is not referenced within text here as an appendix/supplement.<br>- I wonder if rather than having the details in the checklist, you include some/all the relevant detail in the methods text here and indicate that the COREQ checklist will be used for the full study reporting? Otherwise, make sure that references in text are made to any of the supplementary materials.<br>- Reference to the COREQ checklist also prompts me to also wonder what other reporting guideline you intend to use for the other observational aspects of this pre-post (single group) study?<br>- Also, do you have any more information on the development of the interview guide (e.g. was a theory or framework used to identify key question themes) and any pilot testing?	Thank you so much for your feedback. We have added COREQ checklist in text. Changes are done on page no. 12, line no. 17-21.<br><br>We have included SPIRIT (Standard Protocol Items: Recommendations for Interventional Trials) checklist. Changes are done on page no. 6, line no. 7-9.<br><br>The information for interview guide is added on page 12, line no. 7-10.
Patient and public involvement<br>Page 10, line 36 – this sentence is not worded as clear as it could be. How will older adults be informed through the data collection interviews (doesn't seem to make sense - other parts ok). Do you mean involved as study participants in the intervention and outcome assessments (that include interviews and other measures)? How are they involved in the protocol or dissemination of data?	Thank you so much for your feedback. We have revised statement. Changes are done on page no. 13, line no. 15
Discussion<br>Page 10 line 29 - Are there any potential limitations to acknowledge at the outset in this discussion section (aside from the limitations section elsewhere in the manuscript)? Or suggest already what future directions may be, e.g. how do you want to use the quantitative data to inform a future trial design?	Thank you so much for your feedback. We have revised discussion section. Changes are done on page no. 13, line no. 28-34, page no. 14 line no .1 -33
Strengths and limitations section.<br>States 'It is anticipated that the older adults enrolled in this study will find it challenging to wear the device consecutively for seven days. However detailed guidance and instruction will be provided that might ease this	Thanks for your feedback. Changes are done on page 3, line no. 1 -14.

challenge.' Can you please outline what device is meant here, the actigraph or the neck worn device? If mentioning an anticipated challenge with a device in this summary section, I would like to see a clearer link/mention elsewhere in the paper about where this hypothesis/limitation is going to be tested (e.g. is it a question in the interview guide, or use of wear logs for devices), otherwise consider if there is a different key point that is better to highlight about either your study methodology (e.g. single group and no control group (which is probably ok for the stated study feasibility aims) or no longer term follow up beyond 12 weeks about potential sustainability of behaviours) or the intervention itself, in this limitations section.	

Reviewer 2 Comments to Author	Responses
Thank you for the opportunity to review your protocol. The protocol is overall well written and the ideas are clearly articulated. I also commend the authors for focusing on an issue that does not get a lot of research (sedentary behaviour interventions). As a researcher who also does a lot of sedentary behaviour and knowledge translation research I have some considerations to improve the protocol:	Thank you so much for sharing your expertise with us; we appreciate the time you have taken to scrutinise and improve our protocol.
1. The main objective of the study is to "examine the feasibility of a wearable gadget to remind users to break up their sitting time". Some of the outcomes you have selected to assess feasibility (i.e., uptake, data completeness) don't necessarily address your main objective. The purpose of a feasibility study is to answer the question "can this study be done"? There has been several studies on sedentary behaviour where older adults used a wearable device to assess sedentary behaviour - so it's unclear to me why a feasibility study is needed. Is there something specific about this device that requires an additional feasibility study? I wasn't sure if this was actually supposed to be a pilot study (a smaller version of the main study used to test whether the components of the main study can all work together). There is some really good work and guidelines that were published by Dr. Lehana Thabane on feasibility and pilot studies that I recommend you review to help parse out why a feasibility study is needed. Dr. Thabane's paper also goes into the different types of feasibility outcomes	Thank you so much for your feedback. We have revised the section. The suggested reference has also been added. Changes are done on page 5, line number 3-34 and page 6, line no. 1-2.<br><br>Dr. Lehana Thabane reference has been used that guided very well for the study. Changes are done on page 6, line number 7-10.

depending on your research question (e.g., feasibility resource, feasibility process).	
1b. In my own studies, I found certain groups have a lot of mistrust and distrust when using tracking devices. I am wondering how that will be captured in the study? Is there a questionnaire or an exit interview that will take place to capture why they dropped out?	Thank you so much for your feedback. We will investigate drop-out reasons in discontinuing study participants**.**<br><br>Changes are done on page 12, line no. 1-2.
2. I am unsure about how you will identify individuals that are sedentary. According to the 24-hour Canadian guidelines, someone who is sedentary would be an individual that engages in sedentary behaviour for >8 hours per day, has >3 hours of screen time, and does not break up their sedentary behaviour often. You are including individuals who have a self-reported sitting time ≥5 hours; however, this does not mean they are sedentary. A deeper explanation on how you selected this 5 hour cut-off would be helpful. The other problem to consider is that several individuals may accumulate several hours of sedentary behaviour, but don't consider themselves sedentary because it's "active" or "purposeful".	Thanks for your point; it is really interesting. However, as there is no international agreed definition of the "amount" of sedentary time to identify an individual as sedentary, we will just report sedentary time and will not identify the participants as a sedentary group. As the Ekelund harmonised meta-analysis (1) illustrates, there are significant increased risks with increasing ST on all-cause mortality which is how the 5h threshold was derived. In addition, since we have used self-reported sitting time, this is likely an under-estimation, as evident in literature (2).<br><br>1. Ekelund U, Steene-Johannessen J, Brown WJ, Fagerland MW, Owen N, Powell KE, et al. Does physical activity attenuate, or even eliminate, the detrimental association of sitting time with mortality? A harmonised meta-analysis of data from more than 1 million men and women. The lancet. 2016;388(10051):1302-10.<br>2. Dohrn I-M, Gardiner PA, Winkler E, Welmer A-K. Device-measured sedentary behavior and physical activity in older adults differ by demographic and health-related factors. European Review of Aging and Physical Activity. 2020;17:1-11.
3. There is some evidence that seasonal differences affect how sedentary individuals are, with older adults being less sedentary during the warmer months and more sedentary during the colder months. I am wondering if you have considered this variation in sedentary behaviour in your inclusion criteria and in your intervention consideration your intervention takes place in winter and early spring.	Thank you so much for raising this point, it is true that people are more sedentary in colder months therefore it seems to be a better time to intervene because they are going to be at home more and being more sedentary. It is a good time to develop habits which they can translate into behaviour in other months.
4. I noticed gender has not been considered in the protocol. Men and women engage in different sedentary behaviours due to gender norms and roles. How has that been considered in the study?	Thank you so much for raising this point, this is a very excellent point as we have included both genders (males and females) in our study so we can examine in our analysis phase whether they have different effects because men and women engage in sedentary behaviour differently
5. In terms of the intervention, I am having difficulty understanding why this intervention is different from	Thank you so much for this point. Our intervention is different in terms of sample (multi-ethnic older adults and

some previous interventions that have used the same behaviour change techniques. I think one thing to consider is that there is new research that shows most older adults who are healthy are accumulating over 8 hours of sedentary behaviour, but they are also getting up every hour to 1.5 hours. Technically they are breaking up their sedentary behaviour. The second thing that research has shown is that some sedentary behaviours are valued for resting, socializing, and daily functioning, and should not be targeting. Thus not all sedentary behaviours should be targetted, and interventions should move away from targeting total sedentary time to decreasing sedentary behaviours that are not meaningful or have negative health consequences (e.g., consider watching TV versus reading a book for 1 hour). There was a Cochrane review recently published by Chastin that showed none of the behaviour change techniques to target sedentary behavior in older adults were effective - probably because context of sedentary behaviour was never considered (i.e., targeting total sedentary times rather than those behaviours that are bad for health).	age ≥ 65 years) and context (home space) though the behaviour change strategies used are similar to those used in previous research as now mentioned in the introduction section. Changes are done on page 5, line number 3-34 and page 6, line no. 1-2.<br>The other limitation you appropriately raise is that we are not targeting specific sedentary behaviours in our intervention, and we have focused on total sedentary time in the behaviour change intervention, however, the larger effectiveness trial can address this limitation. The purpose of the current intervention is to encourage older adults to do things in non-sedentary or active ways rather than being sedentary. For example, if older adults are sitting and chopping vegetables, they are using their psycho-motor skills however we want them to perform this activity in a standing position or take frequent breaks (sit-to-stand transition) rather than prolonged sitting to maximize health benefits. In a nutshell, as your comments illustrate, it is a really complex area, and it has a drastic impact on health. In this feasibility study with ethnically diverse older adults, who are underrepresented in the research due to various barriers and challenges to their participation, trying to refine or tailor these interventions to apply only in specific circumstances makes engaging them in research even harder. Therefore, in this study we have elected to adopt a simple approach but recognise that further developments are most likely needed as effectively changing targeted SB calls for the most refined interventions that target specific sedentary behaviours to have an effective outcome. Attempting to do this in an initial study with a hard-to-reach population seemed, in our view, a step subsequent to the current one, which was to establish the feasibility of the intervention and the procedure for use with this group of older adults.
6. Could you please provide more detail about how the pamphlets and other components were designed? Were they designed and reviewed by patient partners? What was the process used to design them? What evidence was used to design them?	Thank you so much for your feedback. We have added details. Changes are done on page 8, line no. 6-9
7. Why are wrist worn accelerometers used instead of thigh worn? The accuracy of the wrist worn is a lot lower than the thigh worn accelerometers. There was also a paper that was recently published that suggested using two accelerometers (e.g., thigh and ankle) to improve the accuracy of detecting sit-to-stand movements	Thank you so much for your feedback.<br><br>Wearing a thigh worn device might be considered as exposing or intrusive which could make the OAs in our study feel uncomfortable. In addition, certain religious customs include dress restrictions that emphasise covering certain body parts. Wearing gadgets on the thighs could go against these dress regulations, which would discourage participants from using them. A review has also highlighted that the attitudes of minority ethnic participants towards physical activity were highly

	influenced by religion and cultural dress codes (1-2). Hence wearing an electronic gadget affixed to their thigh may cause discomfort and be considered as culturally inappropriate among ethnic diverse OAs. We therefore chose to use a wrist-worn device for the measuring of pre-post PA. Nevertheless, given the evidence that wrist-worn devices are popular due to their convenience and comfort, this study will use wrist-worn device (3).<br><br>1.Ige-Elegbede J, Pilkington P, Gray S, Powell J. Barriers and facilitators of physical activity among adults and older adults from Black and Minority Ethnic groups in the UK: A systematic review of qualitative studies. Preventive medicine reports. 2019;15:100952.<br><br>2.Koshoedo S, Simkhada P, van Teijlingen E. Review of barriers to engaging black and minority ethnic groups in physical activity in the United Kingdom. Global Journal of Health Science. 2009;1(2):85-96.<br><br>3.Liu F, Wanigatunga AA, Schrack JA. Assessment of physical activity in adults using wrist accelerometers. Epidemiologic reviews. 2021;43(1):65-93.
8. I also recommend collecting medical history, and other outcomes listed in the CIHR equity lens PROGRESS-PLUS.	Thank you so much for your feedback. It is a very good suggestion to add PROGRESS-Plus that includes Place of residence, race/ethnicity/culture/language, Occupation, Gender/sex, Religion, Education, Socioeconomic status, and Social capital and all other potential determinants, for example age, sexual orientation, and disability. However, we have included: age, place of residence, race/ethnicity/culture/language, occupation, gender/sex, religion, education in socio-demographic tool. We are focusing on healthy OAs with no physical and mental impairment to being active therefore we didn't inquire about disability. However, in the larger effectiveness trial the PROGRESS-Plus framework can be used.
9. You mention that you will used reflexive thematic analysis, but the methods don't align with Braun and Clarke's methods of reflexive thematic analysis. I would include a section on your epistemology, positionality, how data credibility will be determined, audit trail, etc. The way the methods are currently written comes across as a thematic context analysis instead of a reflexive thematic analysis.	Thank you so much for your feedback. We have removed, reflexive thematic analysis term and mentioned that we are doing thematic analysis.<br><br>Changes are done on page 12, line no. 27-34
10. The discussion could use more work. I would expand on how this intervention is different from other interventions and why the components you've included	Thank you so much for your feedback. We have revised discussion section. Changes are done on page no. 13, line no. 28-34, page no. 14 line no .1 -33

may be effective and sustainable. I would also include a paragraph about what you predict will happen in terms of the feasibility and limitations of the study and how you plan on overcoming these limitations.	
11. It's not clear to me how the sample size was selected or calculated. A description of why 20 to 25 older adults is needed would be helpful.	Thank you so much for your feedback. We have added references. Changes are done on page no. 6, line no. 9-15.

VERSION 2 - REVIEW

**Reviewer**	**2**
**Name**	**Rodrigues, Isabel**
**Affiliation**	**McMaster University, Rehabilitation Sciences**
**Date**	**24-Sep-2024**
**COI**	

The authors did a great job responding to my revisions. I have no further edits.

VERSION 2 - AUTHOR RESPONSE
